# The Benefit of Non-invasive Ventilation in Motor Neuron Disease

**DOI:** 10.2174/1874306402014010053

**Published:** 2020-12-15

**Authors:** Laura J. Walsh, Desmond M. Murphy

**Affiliations:** 1 Department of Respiratory Medicine, Cork University Hospital, Cork, Ireland.; 2 The HRB- Clinical Research Facility, University College Cork, Cork, Ireland

**Keywords:** Motor neuron disease, Respiratory failure, Non-invasive ventilation, Pulmonary function tests, Forced vital capacity, Nasal

## Abstract

**Background:**

Motor Neuron Disease (MND) is a progressive neurodegenerative disorder leading to respiratory muscle weakness with dyspnoea, morning headaches, orthopnoea, poor concentration, unrefreshing sleep, fatigue and daytime somnolence. Respiratory failure is the primary cause of death in those with MND.

**Methods:**

Although guidelines suggest the use of non-invasive ventilation (NIV) in MND, there lacks clear guidance as to when is the optimal time to initiate NIV and which markers of respiratory muscle decline are the best predictors of prognosis. There have been a number of studies that have found a significant survival advantage to the use of NIV in MND. Similarly, in quality-of-life questionnaires, those treated with NIV tend to perform better and maintain a better quality of life for longer. Furthermore, studies also suggest that improved compliance and greater tolerance of NIV confer a survival advantage.

**Results and Discussion:**

Forced Vital Capacity (FVC) has traditionally been the main pulmonary function test to determine the respiratory function in those with MND; however, FVC may not be entirely reflective of early respiratory muscle dysfunction. Evidence suggests that sniff nasal inspiratory pressure and maximum mouth inspiratory pressure may be better indicators of early respiratory muscle decline. These measures have been shown to be easier to perform later in the disease, in patients with bulbar onset disease, and may indeed be better prognostic indicators.

**Conclusion:**

Despite ongoing research, there remains a paucity of randomised controlled data in this area. This review aims to summarise the evidence to date on these topics.

## 1 INTRODUCTION

Motor Neuron Disease (MND) is a neurodegenerative disorder that leads to progressive muscle weakness and respiratory compromise as a result of the loss of motor neurons in the brain, brainstem, and spinal cord [1-3]. The typical age of onset is in the mid-late 50’s and the disease has a slight male predominance (male: female 2:1). After diagnosis, survival is approximately 3-5 years, with the most common cause of death being respiratory failure [1-4]. The estimated incidence of MND in the UK is 2.76 per 100,000 [5]. Currently, there is no cure for MND, but there have been numerous advances which have improved the survival and quality of life for those with the condition. The only approved drug in MND is riluzole, which works by slowing disease progression and has been shown to prolong survival by 2-3 months [6]. This current review aims to summarise the evidence to date regarding the use of Non-Invasive Ventilation (NIV) in MND and establish the effects it has on the quality of life and survival. There are varying opinions on when it is the best to initiate NIV and which pulmonary function tests are the best predictor of disease progression. Therefore, we will also examine the evidence related to these topics and try to form a clearer consensus on this issue.

## 2 METHODOLOGY

A MEDLINE search of keywords including motor neuron disease or amyotrophic lateral sclerosis, respiratory failure, non-invasive ventilation and pulmonary function tests was carried out in a non-systematic fashion. In particular, papers related to the studies which aimed to assess a potential survival and/or quality of life benefit from the use of NIV in MND were sought. Furthermore, papers related to the timing of NIV initiation, compliance, and tolerance of NIV along with new developments in the treatment of MND were reviewed. These papers were reviewed by the author and if deemed relevant, were included in this study. Studies referenced in these papers which did not show up on the original MEDLINE search were also reviewed and included if relevant.

## 3 MOTOR NEURON DISEASE

MND is a disease that leads to progressive, usually painless, muscle weakness due to both upper motor neuron and lower motor neuron dysfunction [7]. There are various phenotypical subtypes of MND based on the site of disease onset, of which, there are predominantly two, limb onset and bulbar onset. Those with limb onset MND, which constitutes 70% of cases, will generally experience weakness, fasciculations and muscle cramping in the limbs [8]. Patients with bulbar onset MND, which makes up approximately 25% of cases, will often experience dysphonia and dysphagia while the remaining 5% of cases have variable presentations [8]. A diagnosis of MND is based on the fulfilment of three of the El Escorial criteria, which is the gold standard for diagnosis [8]. A poor prognosis in MND is associated with the bulbar onset of disease, frontotemporal dementia, increased age at diagnosis, rapid progression, the presence of c9orf72 gene repeat expansion, a delay in diagnosis and a low Forced Vital Capacity (FVC) at diagnosis [9].

Regardless of the site of onset, however, respiratory muscle dysfunction is a key clinical aspect of disease and disease progression. Evidence of respiratory muscle dysfunction is present in most MND patients at diagnosis [10]. Symptoms of respiratory muscle impairment include dyspnoea and nocturnal hypoventilation, which can lead to morning headaches, orthopnoea, lack of concentration, unrefreshing sleep, fatigue, and daytime somnolence with a resulting negative impact on the patients’ quality of life [10-13]. Dyspnoea is a difficult symptom to quantify in MND and is often not a good indicator of respiratory muscle weakness as patients are often not able to exert themselves to the point of dyspnoea due to limb weakness [14]. In contrast to this, a recent study by Helleman *et al.* [15], evaluating the symptoms of hypoventilation found that dyspnoea and orthopnoea were good predictors of declining respiratory function and the need for NIV use. Interestingly, they found that sleep quality related symptoms, sleepiness and fatigue as reported by MND patients correlated less well with respiratory muscle decline [15]

The primary treatment for these symptoms is the introduction of NIV, which has now been shown to help improve the quality of life and survival in MND [6, 16]. However, the use of NIV in MND is often delayed or not implicated at all due to lack of available resources, lack of physician experience with NIV and a lack of standardised protocols. Bilevel positive airway pressure (BiPAP) ventilation is the most commonly used form of NIV in MND as it most closely replicates physiological conditions, reduces symptoms, improves sleep quality and gas exchange [17-19]. It has been found in a survey of consultant neurologists conducted initially in 2000 and repeated in 2009, that the proportion of people being referred for NIV had increased 2.6 fold while the proportion using NIV had increased 3.4 fold between 2000 and 2009 which only further highlights the necessity for standardised practices when it comes to this intervention [5].

Before commencing NIV, a clear consensus should be reached regarding patients’ wishes for end-of-life decisions. Not every patient will be suitable for NIV. Cognitive impairment, social isolation and rapidly progressive disease may all contribute to unsuitability [5]. MND is often associated with a spectrum of psychiatric conditions which can range from apathy, behavioural and mood changes, disinhibition and in a small number of cases, progression to frontotemporal dementia and as a result, it may exclude NIV as a suitable treatment option [7].

## 4 NICE GUIDELINES REGARDING THE USE OF NIV IN MND

The most recent NICE guidelines on the assessment and management of MND, published in 2016 and updated in July 2019, give some guidance on the use of NIV in those with MND and highlight the signs and symptoms suggestive of respiratory impairment [20]. It is recommended that those with MND have their respiratory function assessed on a regular basis and that other causes of respiratory decline *e.g*. infections should be considered and ruled out before additional treatments are initiated [20]. All patients with respiratory muscle decline should be offered NIV [20]. A discussion on issues such as how often Pulmonary Function Tests (PFTs) should be carried out, frequency of clinic visits, benefits, and risks of NIV, should be made prior to initiation of treatment [20].

**Fig. (1):**
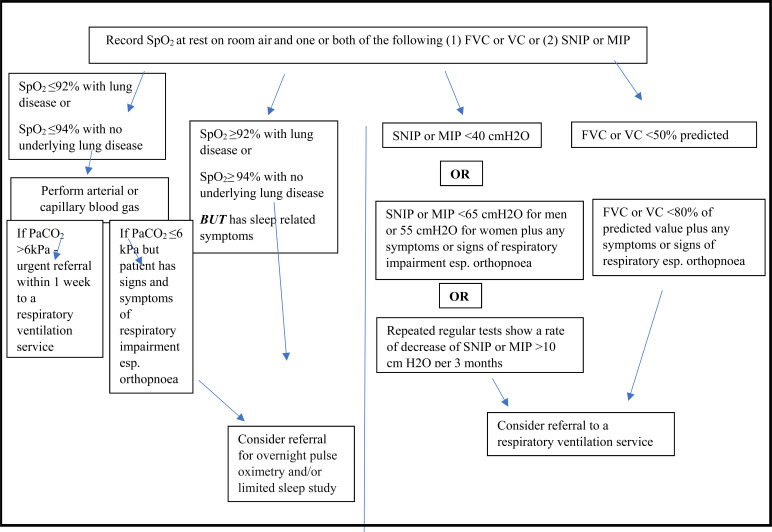
NICE guideline on the introduction of NIV in those with MND. PFTs and SpO_2_ should be monitored at diagnosis and then every 3 months. The results of these should be addressed as above. Any patient with a rapid deterioration in respiratory function should be referred urgently to respiratory ventilation services [20].

At the time of diagnosis or soon after, it is important to establish the baseline respiratory function of each patient by measuring oxygen saturation (SpO_2_) using a pulse oximeter at rest and on room air and that one or both of (1) FVC or Vital Capacity (VC) or (2) Sniff Nasal Inspiratory Pressure (SNIP) and/or Maximal Inspiratory Pressure (MIP) should be measured . Those with severe bulbar impairment or severe cognitive impairment do not have to carry out other PFTs (FVC, at rest, room air, SNIP, MIP) if interfaces are not suitable for the person [20]. Respiratory function testing should be repeated every 2-3 months unless there is a need for more frequent testing. An ABG should be carried out if SpO_2_ is less than 92% with a history of lung disease or less than 94% with no history of lung disease. If a patient has sleep-related respiratory symptoms, then overnight pulse oximetry and/or limited sleep study should be carried out. Patients with an FVC or VC less than 50% predicted or less than 80% predicted and with signs and symptoms suggesting respiratory function impairment or a SNIP less than 40cmH_2_0 or less than 65cmH_2_O (males) or 55cmH_2_O (female) plus signs and symptoms of respiratory muscle decline should be offered NIV (Fig. **1**). It is also recommended that the use of NIV should be commenced initially at night and that treatment hours should be increased as per the patient tolerance [20]. Patient and carer education is also a key aspect of the implementation and success of NIV discussed in this recommendation.

## 5 USE OF NON-INVASIVE VENTILATION IN MND

Although, when exactly to initiate NIV remains debatable, a number of studies have shown that NIV can improve survival and quality of life for those with MND. *Kleopa et al.* [21], carried out a retrospective chart analysis of 122 patients and established that NIV had a positive effect on survival and on reducing the rate of pulmonary function decline, which supported previous smaller studies [22-24]. In this study, participants were reviewed and PFTs were carried out on a 3-monthly basis. BiPAP was offered to patients when the FVC was <50% predicted, when they showed signs of respiratory insufficiency or when the FVC dropped more than 15% predicted in a 3-month period. The patients were then divided into 3 groups. Group one used BiPAP for 4 or more hours per day, group two used BiPAP for less than 4 hours a day and group three refused BiPAP. Overall, the findings showed that those in group one had statistically significant longer survival (35.5 +/- 23.6 months) from diagnosis compared with group three (29.5 +/-12.7 months, p= 0.01), regardless of the type of MND with a potentially higher survival benefit in those with bulbar onset MND after initiation of BiPAP [21]. Furthermore, the rate of decline in FVC tended to be slower in group one and those with bulbar onset after the introduction of BiPAP [21]. This study also highlighted that greater compliance with NIV could also contribute to improved survival.

This survival benefit was further established by Bourke *et al.,* 2006 [25], who carried out the first randomised control trial (RCT) where patients were either assigned to NIV or standard care. In the total study population (n=41), compared with standard care, patients who received NIV (n=22) had considerable improvements in their quality of life, which was sustained for most of the study follow-up period [25]. Median survival for patients treated with NIV was 216 days versus only 11 for those randomised to best supportive care (p=0·0059) [25]. However, this survival benefit did not translate to patients with bulbar onset MND, which contrasts with other studies [21, 26].

There are numerous other studies that demonstrate a survival benefit and improved quality of life following NIV initiation in MND. A systematic review of MND data by Piepers *et al.* [27], found 7 studies that found prolonged survival in patients who tolerated NIV versus those who did not. There were 12 studies in total in this review, and the conclusion was that NIV led to longer survival, improved quality of life, a slower decline in respiratory function and improvement in respiratory symptoms [24, 27-29] (Table **1**).

Similarly, in a prospective study of 22 patients, Bourke *et al.,* 2003 [10] found that the median survival was longer in those who complied with NIV versus those who did not tolerate and that the median rate of decline in VC was slower after starting NIV than before its initiation, without evidence of an early detrimental decline. In addition, there was a more considerable and more sustained improvement in the quality of life after NIV initiation [10]. This study regularly assessed patients using various validated questionnaires, including but not limited to the Short Form 36 (SF36) and the Chronic Respiratory Disease Questionnaire (CRQ). For most domains of quality of life, the peak in improvement was seen 3-5 months after starting NIV and overall, domains of quality of life reflecting sleep disturbance and mental health showed the greatest improvement which were maintained for most of the duration of follow-up and survival despite disease progression [10]. Furthermore, those with moderate to severe bulbar dysfunction had lower rates of compliance and lower improvements of the quality of life but the response to NIV was still clinically useful despite NIV being more difficult to establish in those with bulbar dysfunction [10]. In this study, it was also established that orthopnoea was the best predictor of compliance and was strongly associated with sleep-related symptoms and was the most useful indicator for starting NIV [10, 25].

## 6 INITIATION OF NON-INVASIVE VENTILATION

There has been a long debate about when it is best to initiate NIV in patients with MND. Many earlier studies have taken the value of FVC <50%, predicted as a critical point for starting NIV. However, it has been established in numerous studies that earlier diagnosis of respiratory insufficiency and NIV initiation promote better compliance and therefore have overall better outcomes [16, 30]. In a systematic review of the literature, Miller *et al.,* 2009 [6] showed that early intervention with NIV resulted in 11 months longer survival overall. Furthermore, they established that compliance could improve survival, with those who used the machine for four or more hours per day surviving 3 months longer than those who used it for less than four hours per day [6]. Similarly, in a retrospective observational study with 194 participants, it was shown that very early initiation of NIV, when the FVC>80% was predicted, resulted in more prolonged survival where the crude death rate for very early initiation (FVC>80%) was 35% versus 52.7% (p=0.022) for the groups that started NIV when FVC<80% was predicted over a 3 year period [16].

The benefits of early NIV are also highlighted in a retrospective study of 92 patients where the median time from MND diagnosis to death was significantly longer in those who commenced NIV when FVC ≥65% was predicted compared to those who started NIV when their FVC was <65% predicted (2.7 years vs. 1.8 years) [31]. In addition to this, in a further study with a large cohort of 1034 patients with MND, the median survival of patients with baseline FVC <75% predicted was much shorter than that of patients with baseline FVC >75% predicted, which was independent of medical treatment [32]. Czaplinski *et al.,* also found that a lower FVC at baseline was associated with shorter survival (HR 1.68; 95% CI 1.22 to 2.00; p=0.001) and with more rapid disease progression (1.57; 1.29 to 1.91; p=0.001),which again supports the argument for earlier NIV initiation [32].

There are several hypotheses as to why early initiation of NIV is more beneficial, including the possibility that early NIV may aid in resting a fatigued diaphragm or that earlier intervention may improve lung compliance [31]. Another hypothesis is that by correcting hypoventilation, resulting in hypoxia and muscle acidosis, NIV can improve overall muscle function [31]. Regardless of the pathophysiology, the evidence suggests that the earlier the NIV is started, the better is the outcome. In our institute, we aimed to initiate NIV, usually as an inpatient as soon as possible after the diagnosis is confirmed regardless of muscle function testing and symptoms.

## 7 COMPLIANCE AND TOLERANCE

Compliance with and tolerability of NIV are the key factors in determining the success of treatment. Overall, it is felt that people who are more compliant and can better tolerate NIV gain the most significant overall benefit and this has been shown in a number of studies. Bourke *et al.,* (2003) found that the survival benefit they observed in their small (n=22) prospective study correlated with NIV compliance (p0.016) [10]. Furthermore, in the same study, in multivariant analysis, compliance with NIV was the only factor that correlated with the quality of life [10]. Kleopa *et al.,* (1999) also found that there was a significant survival benefit to those who had good NIV tolerance *i.e.* >4 hours per night, versus those who did not tolerate and therefore did not use NIV [21]. They also found in this study that the area of onset of MND *i.e.* limb or bulbar, did not affect tolerance [21] while other studies have found that bulbar symptoms have been associated with poor NIV tolerance and therefore reduced benefit [25].

Compliance can be improved by early initiation of NIV [30]. Furthermore, gradually increasing NIV use from nocturnal use initially to gradually increasing use during the day can also help improve tolerance and hence compliance [16]. The presence of orthopnoea is also a strong predictor of benefit and compliance with NIV similar to the young age and preserved upper limb strength [6, 15]. Noncompliance can become an issue in those with frontotemporal dysfunction, and as mentioned in those with bulbar symptoms [6].

## 8 PULMONARY FUNCTION TESTING IN MND

It has long been established that the rate of decline of the respiratory muscle function is related to mortality [33]. However, a debate remains as to which particular measurement of respiratory function is the best indicator of this decline. Previously, the FVC was found to be a specific and sensitive indicator of disease progression in MND and a better predictor of death than neuromuscular scores [32, 34]. Furthermore, the FVC has been found in a number of studies to be the factor that best correlated with respiratory symptoms [3, 33, 35]. Currently and as previously stated, earlier introduction of NIV leads to greater compliance and, therefore, more overall benefits in terms of quality of life and survival. However, in this case, the FVC may then not be the best indicator of declining respiratory function as FVC may not decline until there is already profound muscle weakness, which is due to the sigmoid relationship of the lung pressure curves [36-38]. Also, patients with bulbar weakness often cannot form an adequate mouth seal and so recorded FVC values may not be reflective of true muscle weakness [36]. Therefore, the use of other pulmonary function tests, which may better indicate early respiratory involvement, has been studied with good outcomes.

Sniff Nasal Inspiratory Pressure (SNIP) may be a more accurate prognostic indicator of respiratory muscle weakness, but data is lacking on its usefulness in determining when to initiate NIV [39]. In order to perform SNIP, a small tube is placed in the nostril and with the other nostril plugged, the maximum sniff from relaxed to end-expiration is measured [39]. In one prospective Irish study of 98 patients, the SNIP was found to correlate well with transdiaphragmatic pressures and was more likely to be performed later in the disease [36]. It was established that on the patient's last visit before death, SNIP was more likely to be recorded or reproduced compared with FVC and another measurement of respiratory function, the maximum Mouth Inspiratory Pressure (MIF), both of which being more difficult to perform as the disease progresses [36]. Only 4% of these patients were unable to perform SNIP at this visit, while 14% could not perform FVC and 19% could not perform MIF [36]. A SNIP <40cm H_2_O was associated with a hazard ratio for death of 9.1 (CI 4-20.8, p< 0.001), while when the FVC fell below 50%, the hazard ratio for death became 5.66 (CI 2.73-11.73, p< 0.001) [36]. Interestingly, among patients with a SNIP <40cm H_2_O, 66% had an FVC >50% and the hazard ratio for death in this incidence was 13.6 (CI 3.1 -54.7, P<0.001) [36]. Overall, studies would conclude that SNIP measurements can better predict respiratory muscle function as it linearly declines with disease progression [40, 41], can be better performed in advanced disease, is a reliable predictor of mortality and can better indicate earlier respiratory muscle dysfunction than FVC [36, 40, 42, 43]. Furthermore, it does not require a good mouth seal, which may be lost in bulbar onset MND [40].

In addition to SNIP, MIP has also been shown to be more sensitive than FVC at predicting early respiratory muscle decline in neuromuscular disorders [44]. However, caution should be used in interpreting MIP and maximum expiratory pressure (MEP) data, as low values may result from a lack of motivation and poor efforts [45]. Furthermore, MIP is often considered to be more difficult to perform than SNIP in patients with MND, with increasing difficulty in performing MIP as the disease progresses [37, 46-48]. Therefore, Janssen *et al.* [45], suggest to perform SNIP and MIP simultaneously as this may decrease the risk of over-diagnosing respiratory muscle dysfunction in neuromuscular diseases and both are considered more sensitive than FVC at detecting respiratory muscle involvement in early MND as they are impaired earlier than the FVC. However, the feasibility of both tests has been questioned in subjects with bulbar onset MND [40, 42].

A further pulmonary function test to consider is the Peak Cough Flow (PCF), which again has been shown to be effective, but like SNIP, it lacks adequate data in its utility to determine the timing of NIV initiation [39]. PCF is measured by performing a maximum inspiration followed by a forceful cough with the lips sealed around a tube [39]. It has been shown that the PCF has the greatest predictive value for NIV initiation when compared with FVC, MIP and MEP and SNIP [39]. Despite this, it has been established that measurements such as SNIP and PCF, although proven to be more reliable than FVC at predicting respiratory muscle decline, are less likely to be performed in a clinical situation [5, 39].

## 9 OTHER TREATMENTS IN MND

Other interventions which can improve survival in MND include the use of cough assist devices, which can help clear excessive secretions and therefore prevent infections [7]. Mechanical insufflation-exsufflation (MIE) devices can help produce adequate cough, therefore preventing accumulation of secretion [8]. Having said that, a 2013 Cochrane Review demonstrated that the use of MIE devices augmented peak cough expiratory flow but did not demonstrate superiority over other mucus clearing techniques, including manually assisted cough [49].

The COVID-19 pandemic has changed the way in which we practice medicine and certainly, even before the outbreak, research was underway to evaluate how people with MND could be remotely monitored safely to avoid the exhausting experience of attending outpatients clinics when not needed. Helleman *et al.* [15], developed and tested a new MND questionnaire called the MND dyspnoea scale (MND-DS) which they proved was more beneficial when it comes to the monitoring of the respiratory muscle decline in MND patients over the respiratory domain of the amyotrophic lateral sclerosis functional rating scale (ALS-FRSr), which is a validated tool for the assessment of symptoms in patients with MND. They found that the MND-DS correlated well with respiratory muscle decline and that it can be used remotely to monitor respiratory function in MND patients in between clinics. Added to this, Ando *et al.* [50], describe a telemonitoring system they developed for use in MND patients on NIV using a Careportal^R^ device which allows clinicians to monitor any changes in patients’ symptoms, nocturnal oxygen saturations and also allows communication between the patient and clinician through the device. There have been a number of studies which studied the benefit of teleconferencing and telemonitoring in MND and in monitoring NIV compliance [51-53], but Ando *et al.,* appear to be the first who shared the subjective experience of patients using a telehealth system [50]. Overall, this study found that there was a positive physical and psychological benefit to the patients’ health status being monitored regularly and patients found reassurance in this [50]. It reduced the need for unnecessary hospital appointments. It helped to improve the self-awareness of the patients’ condition and patients also appreciated the communication with clinicians through the device portal.

Remote monitoring, however, also requires a remote assessment of PFTs in order to objectively assess patients’ wellbeing. Geronimo *et al.* [54], evaluated the use of remote testing of PFTs in 40 patients to establish if remote PFT testing was as reliable and accurate as PFTs in the clinical setting. They found that there was a high correlation between remote PFT testing and standard PFT testing and that patients appreciated the convenience of the method [54]. It was also felt that this could allow for closer monitoring of PFTs and could potentially identify rapidly deteriorating PFTs faster and improved outcomes for these patients [54]. The only disadvantage of this study is that it excluded those with bulbar dysfunction. Overall, these studies suggest that remote monitoring of those with MND on NIV is possible and convenient for patients. Now, in this current climate, it does necessitate the need for MND clinics to consider this as an option.

## CONCLUSION

NIV has a role in the treatment of patients with MND as it has benefits when it comes to survival and improvements in quality of life. A large part of this review focuses on the best measure of lung function that can be used to best predict respiratory muscle decline. However, there are other methods currently under investigation, which may prove more effective. Chest ultrasound is used to identify pleural and parenchymal abnormalities in several respiratory diseases and to assess diaphragmatic function and may additionally have a role in those with MND [8]. The test is carried out by measuring diaphragmatic thickness and movement during breathing [8]. Recently, diaphragmatic ultrasound has been validated in mechanically ventilated patients and other studies conducted in patients with MND with and without bulbar dysfunction have shown that ultrasound can be applied to the diaphragm in order to assess its function with good reliability [8]. More research needs to be carried out in order to fully evaluate this method, but certainly, early results look promising.

A Cochrane review of mechanical ventilation for amyotrophic lateral sclerosis/MND (2018) aimed to review evidence on the use of NIV in MND and in particular, its benefits on survival and quality of life [55]. It concluded that there was moderate evidence that NIV improved survival in MND and low-quality evidence that it improved quality of life. However, it should be noted that only one RCT was included in this review, therefore, a metanalysis could not be performed as other studies did not meet inclusion criteria. The Cochrane review does acknowledge that the results of the study by Bourke *et al.,* (2006), which has been reported in this present review, are significant enough that further RCTs on NIV versus standard of care are likely to be unethical. Overall, an established survival benefit and a pronounced benefit to patients’ quality of life with the use of NIV have been proven in a number of studies. However, the majority of studies involved small patient numbers or short follow-up times and class I evidence on the subject is lacking. The establishment of specific guidelines may lead to better implementation of NIV in centres where neurologists and respiratory physicians do not co-manage MND patients.

## Figures and Tables

**Table 1:** Summary of studies. Study author, type of study, number of participants, main aim of study and the primary result of each study is mentioned. These studies relate to papers where a survival benefit and/or a quality of life benefit from the use of NIV in MND, when best to initiate NIV in MND, compliance and tolerance of NIV in MND and the measurement of pulmonary function tests in MND was discussed.

** Author, Year (reference no) **	** Type of Study **	** N **	** Aims of study **	** Summary of Results **
Aboussouan *et al.* 1997 [24]	Prospective Observational study	39	To assess if there is a survival benefit in those who tolerate BiPAP versus those who do not tolerate BiPAP	18 patients tolerated NIV. The rate of death was 3.1-fold greater in intolerant versus those who tolerated BiPAP
Aboussouan *et al. 2001* [29]	Prospective Observational study	60	Effect of NIV on survival and quality of life	Improved survival and better compliance in those that tolerated NIV
Bach *et al.* 2002 [28]	Retrospective Observational study	101	Assess if survival benefit to the use of NIV in those with ALS	A 14-17-month improvement in survival in those with ALS treated with NIV
Berlowitz *et al.* [24]	Retrospective cohort analysis	929	Assess the effect of NIV on survival, whether MND phenotype influences survival and if NIV affects the rate of pulmonary function decline	Median tracheostomy free survival 28 months in those who received NIV compared with 15 months in those without NIV and a slower rate of PFT decline with NIV
Bourke *et al.* 2003 [10]	Prospective Study	22	Assess the impact of NIV on quality of life	A survival and quality of life benefit is strongly related to NIV compliance
Bourke *et al*. 2006 [25]	Randomised control trial	41	Assess quality of life and survival in patients treated with NIV compared with standard care	Improved quality of life and survival in the group treated with NIV
Czaplinski *et al.* 2006 [32]	Retrospective cohort analysis	1034	Assessment of FVC at baseline to time to progression of 20 points in Appel ALS score or tracheostomy free survival	Higher FVC at diagnosis is associated with longer survival and slower progression of FVC decline
Kleopa *et al*. 1999 [21]	Retrospective Observational study	122	The effects of BiPAP on survival and the progression of pulmonary function tests with ALS	BiPAP improved survival and slowed the progression of pulmonary function decline
Lechtzin *et al.* 2007 [31]	Retrospective study	92	Assessment of tracheostomy free survival from diagnosis	Improved survival with early initiation of NIV
Miller *et al.* 2009 [6]	Systemic literature review	142 articles	Update on the management of ALS	Provision of several recommendations to improve the management of ALS
Morgan *et al.* 2005 [36]	Prospective observational study	98	Prognostic value of FVC, MIF and SNIP over 3 years	SNIP correlated well with transdiaphragmatic pressures, SNIP was more likely to be assessed pre death and was more sensitive and specific at predicting6 month mortality
Piepers *et al.* 2006 [27]	Systemic review	12 studies	Assessment of survival, quality of life and rate of respiratory function decline in those treated with NIV compared with those that didn’t tolerate NIV	Seven studies showed a survival benefit in those treated with NIV versus those who didn’t tolerate it. There was also an improvement in the quality of life and a slower rate of decline of respiratory function compared with those who didn’t tolerate NIV
Pinto *et al*. 1995 [23]	Prospective controlled trial	20	To assess the utility of BiPAP in improving survival in patients with ALS	Overall, significant improvement in survival with patients treated with BiPAP (p<0.004)
Pinto *et al.* 2003 [30]	Prospective comparative study	42	Assess survival in MND patients treated with NIV	Total survival was longer in those treated with NIV
Sherman *et al.* 1994 [22]	Observational Study	170	Assessment of Respiratory care in ALS	There was a significant survival benefit in those treated with BiPAP
Vitacca *et al.* 2018 [16]	Retrospective Observational study	194	Aim to assess would very early initiation of NIV have an impact on survival	Very early commencement of NIV had a significant improvement in survival

## LIST OF ABBREVIATIONS

MND: = Motor Neuron Disease
NIV: = Non-invasive Ventilation
FVC: = forced Vital Capacity
Bilevel Positive Airway Pressure: = BiPAP
NICE: = The National Institute for Health and Care Excellence
PFTs: = Pulmonary Function Test
VC: = Vital Capacity
SNIP: = Sniff Nasal Inspiratory Pressure
MIP: = Maximal Inspiratory Pressure
ABG: = Arterial Blood Gas
SF36: = Short Form 36
CRQ: = Chronic Respiratory Disease Questionnaire
